# Expression levels of the microRNA maturing microprocessor complex components; Drosha, Dicer, and DGCR8 in PBMCs from ankylosing spondylitis patients

**DOI:** 10.31138/mjr.28.2.80

**Published:** 2017-06-27

**Authors:** Zeinab Tabrizi, Reza Mansouri, Saeed Aslani, Ahmad Reza Jamshidi, Mahdi Mahmoudi

**Affiliations:** 1 Rheumatology Research Center, Tehran University of Medical Sciences, Tehran, Iran,; 2 Immunology Department, Shahid Sadoughi University of Medical Sciences (International Campus), Yazd, Iran

**Keywords:** Drosha, Dicer, DGCR8, Ankylosing spondylitis

## Abstract

**Objective/Aim:**

Two major enzymes in the microRNA maturation process, Dicer and Drosha, as well as DGCR8, the assistant of Drosha, function in the microprocessor complex. In this survey, the mRNA expression profiles of Drosha, Dicer, and DGCR8 in peripheral blood mononuclear cells (PBMCs) from ankylosing spondylitis (AS) patients and healthy controls were measured

**Methods:**

Forty patients with AS and 40 age and gender matched healthy individuals were included in the study. PBMCs were separated, total RNA content of the cells was isolated, and first strand cDNA was synthesized. Quantitative analysis was performed through real-time PCR using the SYBR Green gene expression master mix.

**Results:**

AS cases expressed the Drosha mRNA almost equal to that of healthy controls (Fold Change= −0.94; P= 0.200). However, both Dicer and DGCR8 mRNA expressions were downregulated in patients relative to healthy subjects (Fold Change= −0.54 and −0.60; P= 0.002 and 0.004, respectively).

**Conclusion:**

Our results suggest that downregulation of miRNA maturation components, namely Dicer and DGCR8 may be contributing in the pathogenesis procedure of AS.

## INTRODUCTION

MicroRNAs (miRNAs), a group of non-coding RNAs, are post-transcriptional regulators which are involved in many biological procedures, including development, differentiation, proliferation, and apoptosis. ^1^ They are substantial gene expression regulators that control both physiological and pathological processes such as cancer, auto-immune diseases, and chronic inflammatory conditions. ^2^ miRNAs synthesis initiates by transcribing miRNA genes into primary miRNA (pri-miRNA) transcripts through either RNA polymerase II or RNA polymerase III. ^3^ As the hairpins are formed in pri-miRNA, it is identified by a nuclear protein known as DiGeorge syndrome critical region gene 8 (DGCR8 or Pasha) which is linked with Drosha protein, an enzyme that cuts RNA to develop the microprocessor complex. ^4^ In this complex, the catalytic RNase III domain of Drosha, which is oriented by DGCR8, releases the hairpins from pri-miRNAs by cleaving approximately eleven nucleotides from the hairpin base to produce pre-miRNAs. ^5^ After that, the pre-miRNA with 70–90 nucleotide length is transported to the cytoplasm through exportin-5 for further processing. In the cytoplasm, Dicer, which holds RNase III enzyme activity, processes the pre-miRNA to create mature miRNA with desired function. ^6^ Therefore, it seems that the role of Drosha, Dicer, and DGCR8 as the major miRNA biogenesis constituents is inevitable.

Ankylosing spondylitis (AS) is a type of arthritis with chronic inflammation in the spine and sacroiliac joints, that is commonly defined by pain and stiffness at the spine and other peripheral joints. ^7–9^ Given that AS is a multifactorial disease, it is a consensus nowadays that both environmental factors and genetics are involved in the initiation and perpetuation of the disease. ^10,11^ However, the exact etiology underlying the pathogenesis of AS remains obscure. Human leukocyte antigen (HLA)-B27 has been identified to be vigorously associated with AS but accounts for only a part of the overall risk for it, implying to the involvement of other genes in the development of the disease. ^12–15^

The contributory role of miRNAs to the pathological conditions involving the immune system, like autoimmunity has been addressed by numerous studies. Even though downregulation or upregulation of several miRNAs has been shown in this disease, their involvement in the etio-pathology of AS has not been surveyed extensively.16 Three miRNAs, miR-16, miR-221, and let-7i, were upregulated in T cells obtained from AS patients. Upregulation of reported miRNAs were found to be correlated positively with the disease activity. ^17^ Moreover, miR-29 has been known as a useful diagnostic marker for new bone formation in AS and might be a promising therapeutic tool. ^18^ Recently, studies have concentrated on the involvement of miRNAs in AS disease, suggesting that miRNAs may be involved in the development of AS. Considering the controversial expression level of miRNAs in peripheral blood cells of AS patients, and noting that expression levels of Drosha, Dicer, and DGCR8 genes have not been investigated in AS, we decided to measure the transcript level of these three primary components of miRNA bio-synthesis machinery in AS patients.

## MATERIALS AND METHODS

### Study subjects

The patients were recruited from Rheumatology Research Center, Shariati Hospital, Tehran, Iran, and diagnosis of AS was carried out according to the modified New York criteria. ^19^ Intensity of the disease and functional limitations were measured by bath ankylosing spondylitis disease activity index (BASDAI), ^20,21^ bath ankylosing spondylitis functional index (BASFI), ^21,22^ and bath anky-losing spondylitis metrology index (BASMI). ^20,21^ Forty AS patients and 40 healthy controls (age and gender matched) were included in the study. Healthy control volunteers did not have autoimmune disease neither themselves nor their immediate family members. Patients had not received immunomodulatory therapy for at least 3 months before they were entered in the study. The Human Research Ethics Committees from Tehran University of Medical Sciences sanctioned this study. All the participants signed the informed consent form. From each subject, 10 ml of blood was obtained in EDTA-anticoagulated and ESR blood collection test tubes by venipuncture.

### PBMC isolation and RNA extraction

For isolating the PBMCs from peripheral blood, the Ficoll-Hypaque density gradient centrifugation approach was exploited. High Pure RNA Isolation Kit (Roche, Germany), according to manufacturer’s manuals, was used for total RNA extraction. To evaluate the yield and purity of extracted RNA, NanoDrop spectrophotometer (2000c, Thermo Fisher Scientific, USA) at 260/280 nm absorbance was applied.

### cDNA synthesis

After RNA extraction, synthesis of first strand complementary DNA (cDNA) was done using the Transcriptor First Strand cDNA Synthesis Kit (Roche, Germany). In brief, to synthesize cDNA in a final volume of 20 μl, 1 μg of RNA was added in a micro tube, and mixed with 2 μl of primer (random hexamer) and adequate RNase-free H _2_ O; and then incubation was done at 65°C for 10 minutes. Following micro tubes were chilled on ice, and RNase inhibitor 0.5 μl, reaction buffer 4 μl, dNTP mix 2 μl, and reverse transcriptase enzyme 0.5 μl were added to each micro tube. Afterwards, each tube was incubated at 25°C for 10 minutes and followed by 50°C for 60 minutes; and the reaction was finished after heating at 85°C for 5 minutes.

### Quantitative real-time polymerase chain reaction

Primers used in this study were adopted from the previously published works by Sand et al, in which the transcript levels of Drosha, Dicer, DGCR8, and RNA induced silencing complex (RISC) components in epithelial skin cancer were determined. ^23,24^ In order to evaluate the specificity, primers (Drosha, Dicer, DGCR8, and RPL38 as a housekeeping gene) were checked using the Basic Local Alignment Search Tool (BLAST) on the NCBI website. ^25^ Refer to **Table 1** for the primer’s features with more details.

**Table 1.** Primers used for real-time gene expression of Drosha, Dicer, and DGCR8.

**Target genes**	**Sequence**	**Amplicon size (bp)**	**Tm (°C)**
Drosha F	5′-CATGTCACAGAATGTCGTTCCA-3′	115	58.4
Drosha R	5′-GGGTGAAGCAGCCTCAGATTT-3′	115	59.8
Dicer F	5′-TTAACCTTTTGGTGTTTGATGAGTGT-3′	94	58.5
Dicer R	5′-GGACATGATGGACAA TTTTCACA-3′	94	57.1
DGCR8 F	5′-GCAAGATGCACCCACAAAGA-3′	93	57.3
DGCR8 R	5′-TTGAGGACACGCTGCATGTAC-3′	93	59.8
RPL38 F	5′-TCACTGACAAAGAGAAGGCAGAGA-3′	88	61
RPL38 R	5′-TCAGTGTGTCTGGTTCATTTCAGTT-3′	88	59.7

Quantitative analysis was carried out by real-time PCR using the SYBR Green gene expression master mix (Takara Bio, Inc.) and StepOnePlus real-time PCR system (Applied Biosystems, Foster City, CA, USA). Each reaction mixture contained a total volume of 20 μl (master mix 10 μl, cDNA 2 μl [5ng/ml], forward and reverse primer 1 μl of each, and H _2_ O 6 μl). The conditions of quantitative real-time PCR were: first, 95°C for 30 seconds, 40 cycles in 95°C for 15 seconds, and finally 60°C for 1 minute. To calculate the relative gene expression, comparative C _T_ method was used, as previously elucidated by Livak and Schmittgen. ^26^ Relative transcript levels of the target mRNAs were normalized versus to the corresponding housekeeping gene, namely RPL38 mRNA transcript level. Ultimately, the relative mRNA expression level for each sample was calculated by the equation below, for simplicity of visualization: relative mRNA expression= (2 ^−ΔCt^ ) × 100.

### Statistical Analysis

Analysis of data was fulfilled via SPSS software version 21 (SPSS, Chicago, IL, USA). To assess the normal distribution of the scale variables, the Kolmogorov–Smirnov test was used. The independent sample *t* -test was used to compare groups with continues variables. The Graph-Pad Prism version 5.00 software (GraphPad Software, La Jolla California USA, www.graphpad.com) was applied to draw the graphs. Scale data were expressed as mean ± standard deviation (SD). A *P* -value < 0.05 was set to be statistically significant.

## RESULTS

Study population was comprised of 40 AS patients (29 males and 11 females) with the mean age of 39.8 ± 11.49 and 40 healthy individuals (30 males and 10 females) with the mean age of 38 ± 9.39. ESR level of the patients and healthy controls were 35 ± 18.24 and 4.4 ± 3.82, respectively. BASDAI, BASFI, and BASMI scores of the AS patients were 4.44 ± 2.13, 2.9 ± 2.08, and 4.56 ± 1.9, respectively ( **Table 2** ).

**Table 2.** Demographic specifications of ankylosing spondylitis patients and healthy controls.

**Property**	**AS patients**	**Healthy controls**
Male	29	30
Female	11	10
Age	39.8 ± 11.49	38 ± 9.39
ESR	35 ± 18.24	4.4 ± 3.82
BASDAI	4.44 ± 2.13	-
BASFI	2.9 ± 2.08	-
BASMI	4.56 ± 1.9	-

**Figure 1:**
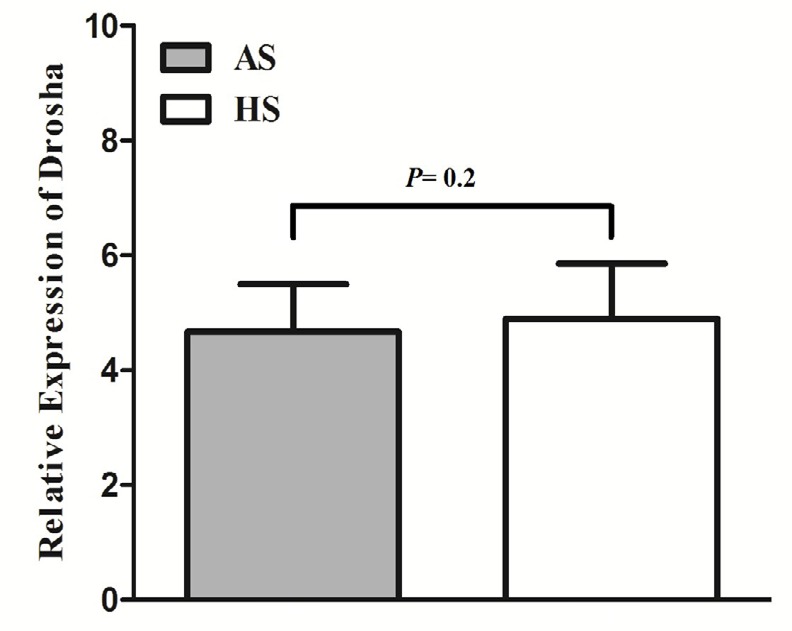
Relative expression of Drosha mRNA in PBMCs from AS patients and healthy subjects is illustrated through Bar graph. AS patients did not express Drosha mRNA significantly different than control group (AS: Ankylosing spondylitis, HS: Healthy subjects).

**Figure 2:**
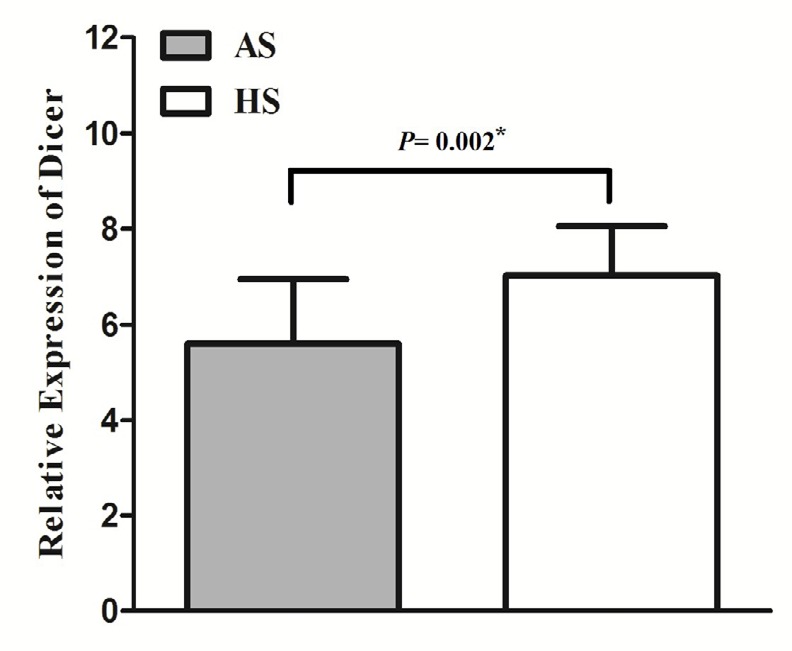
Relative expression of Dicer mRNA in PBMCs from AS patients and healthy subjects is illustrated through Bar graph. Expression of Dicer mRNA was significantly downregulated in AS patients compared with healthy subjects (AS: Ankylosing spondylitis, HS: Healthy subjects).

**Figure 3:**
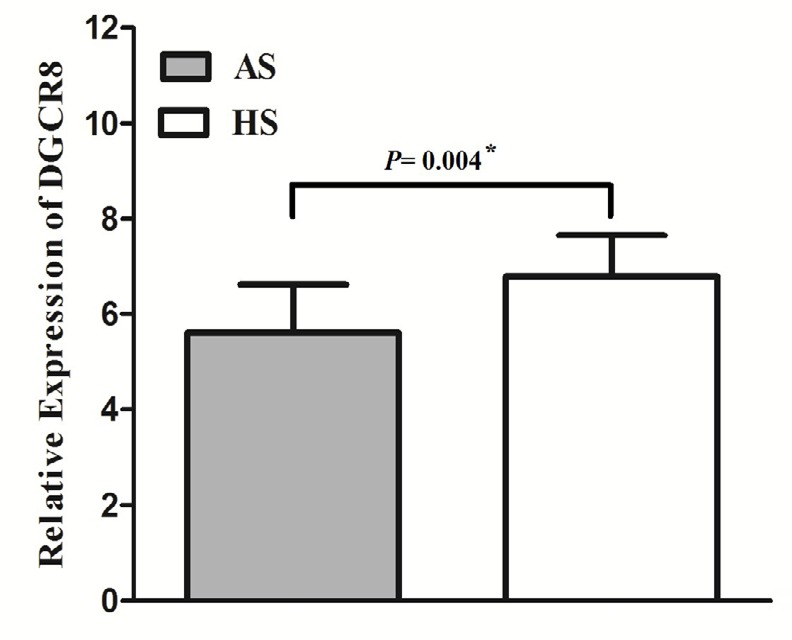
Relative expression of DGCR8 mRNA in PBMCs from AS patients and healthy subjects is illustrated through Bar graph. Expression of DGCR8 mRNA was significantly downregulated in AS patients compared with healthy subjects (AS: Ankylosing spondylitis, HS: Healthy subjects).

We did not observe statistically significant difference (Fold change= −0.94; *P* = 0.200) in the expression level of Drosha between AS patients and healthy control groups ( **Table 3 , Figure 1** ). However, PBMCs from AS patients expressed the Dicer mRNA lower (0.54 times downregulated; *P* = 0.002) than healthy subjects ( **Table 3 , Figure 2** ). Also, downregulation of DGCR8 mRNA ( **Table 3 , Figure 3** ) was seen in AS patients compared with healthy controls (Fold change= −0.60; *P* = 0.004).

**Table 3.** Fold changes of Drosha, Dicer, and DGCR8 expressions in patients with ankylosing spondylitis (AS) in comparison to healthy subjects (HS).

**Target Gene**	**Fold Change (AS vs. HS)**	**P-value**
Drosha	−0.94	P= 0.200
Dicer	−0.54	P= 0.002
DGCR8	−0.60	P= 0.004

Given the importance of BASDAI, BASFI, and BASMI indices in screening the clinical manifestations of AS patients, we tried to evaluate the correlation of those with mRNA expression levels of target genes ( **Table 4** ). Relative expression level of Drosha did not correlate with BASDAI ( *P* = 0.167, *r* = −0.203), BASFI ( *P* = 0.318, *r* = −0.147), and BASMI values ( *P* = 0.883, *r* = −0.022) of AS patients. As such, none of the BASDAI ( *P* = 0.447, *r* = 0.122), BASFI ( *P* = 0.596, *r* = −0.085), and BASMI ( *P* = 0.156, *r* = −0.226) scores of AS patients correlated with relative expression level of Dicer. Furthermore, relative expression of DGCR8 did not show correlation with BASDAI ( *P* = 0.512, *r* = 0.110), BASFI ( *P* = 0.954, *r* = −0.010), and BASMI ( *P* = 0.341, *r* = 0.159) scores of AS patients.

**Table 4.** The correlations of the expressions of Drosha, Dicer, and DGCR8 mRNAs in PBMCs from AS patients with BASDAI, BASFI, and BASMI (r; pearson’s correlation coefficient).

**Target genes**	**BASDAI *r* (*P*-Value )**	**BASFI *r* (*P*-Value )**	**BASMI *r* (*P*-Value )**
Drosha	−0.203 (*P*= 0.167 )	−0.147 (*P*= 0.318 )	−0.022 (*P*= 0.883 )
Dicer	0.122 (*P*= 0.447 )	−0.085 (*P*= 0.596 )	−0.226 (*P*= 0.156 )
DGCR8	0.110 (*P*= 0.512 )	−0.010 (*P*= 0.954 )	0.159 (*P*= 0.341 )

## DISCUSSION

Application of epigenetics to autoimmune diseases, and especially AS, is in its infancy and requires new hypotheses. ^27^ miRNAs, as a classification of epigenetics modifications, have been associated with AS by a few researches and is a new trend to gain a bigger insight into the etiopathology of this disease. Hopefully, an exhaustive knowledge of miRNA implication could help to recognize novel therapeutic targets and biomarkers for this disease. ^16^ In recent years, dysregulation of miRNAs has been reported in AS. miR-16, miR-221, and let-7i have been observed to be upregulated in AS patients, among which let-7i and miR-221 were found to be correlated positively with Bath Ankylosing Spondylitis Radiology Index (BASRI) of lumbar spine. The study suggested that the increased expression of let-7i in AS T cells contributed to the immunopathogenesis of the disease, which is probably mediated by enhancing the Th1 (IFN-γ)-associated inflammatory response. ^17^ Moreover, let-7i was shown to regulate Dicer expression and constitute a negative feedback loop. In other words, overexpression of let-7 significantly declined the expression of Dicer at both the protein and mRNA levels. Thus, upregulation of let-7 maybe the cause of Dicer downregulation in a similar way in AS. ^28^ As shown by Huang et al. 2014, miR-29 that directly targets the Dkk-1 mRNA, an antagonistic inhibitor of the WNT signaling pathway, had overexpression in the PBMCs from AS patients compared with rheumatoid arthritis (RA) patients as well as healthy subjects, ^29^ suggesting that miR-29a is a useful diagnostic marker for new bone formation in AS and might be a promising therapeutic agent in the future. ^18^ Furthermore, miR-146a can act as a regulator to prevent an overstimulated inflammatory state by impairing NF-κB activity. As a result, receptor activator of NF-κB ligand (RANKL) and pro-inflammatory cytokines contribute to the development of AS. Therefore, lower expression of miR-146a provides prolonged release of inflammatory cytokines in AS. ^30^ As detected by Qian BP et al in 2016, the expression level of miR-155 that may serve as a novel complementary bio-marker for AS, is suggested to be associated with disease activity (BASDAI) and the severity of thoracolumbar kyphosis in AS patients. ^31^

On the other hand, the role of miR-155 in the differentiation and function of regulatory T cells (Tregs) has been reported. In this context, several studies are indicated that conditional deletion of Dicer or Drosha in Tregs culminated in lethal inflammatory autoimmune setting, is mediated by defective development or function of Tregs. Therefore, reduced expression of Dicer and Drosha may contribute to impaired development of Tregs, implying to the role of miRNAs in the biology of Tregs and autoimmunity. ^32^

Accordingly, investigation of miRNA processing components including Drosha, Dicer and DGCR8 is of importance to answer the questions around aberrant expression of miRNAs in AS. Several studies not only have tried to realize the expression profile of miRNA microprocessing compartments in various disorders but they also reported the dysregulated expression profiles of these three molecules in cancerous and autoimmunity conditions. Taken together, miRNAs play a role in the pathogenesis of AS. ^23,33–26^

Here, we observed dysregulated expression level of these components in AS patients, however Drosha did not show significantly different expression level between AS patients and healthy controls. Furthermore, Dicer and DGCR8 were downregulated 0.54 and 0.60 times in patients, respectively. Moreover, none of the Drosha, Dicer, and DGCR8 expression levels correlated with disease activity indices as measured through BASDAI, BASFI, and BASMI. Even though premature, our findings favor the hypothesis that miRNAs are involved in the pathogenesis of AS by dysregulated expression levels of microRNA processing enzymes.

Considering all the facts, our results, as a first time reporting the expression level of miRNA processing machinery molecules in AS, suggest that downregulation of the major miRNA machinery components, Dicer and DGCR8, provides new evidence for plausible involvement of dysregulated miRNAs in the AS development. These molecules are mandatory to be further investigated considering the other miRNA biogenesis components. Comprehensive studies may lead to introduction of new disease biomarkers for assessing possible therapeutic approaches and disease activity that may facilitate the identification of novel therapeutic targets in the future.
